# Development of a MassARRAY Genotyping Platform and Its Clinical Application for Venous Thromboembolism Risk Assessment in Thai Patients

**DOI:** 10.3390/medsci13040282

**Published:** 2025-11-24

**Authors:** Dollapak Apipongrat, Chonlada Laoruangroj, Oytip Nathalang, Pasra Arnutti, Montalee Theeraapisakkun, Wittawat Chantkran

**Affiliations:** 1 Department of Pathology, Phramongkutklao College of Medicine, Bangkok 10400, Thailand; dollapak.a@gmail.com; 2 Division of Hematology, Department of Medicine, Phramongkutklao Hospital, Bangkok 10400, Thailand; amchon25@gmail.com; 3 Graduate Program in Biomedical Sciences, Faculty of Allied Health Sciences, Thammasat University, Pathumthani 12120, Thailand; oytipntl@hotmail.com; 4 Department of Medical Technology, Faculty of Allied Health Sciences, Pathumthani University, Pathumthani 12000, Thailand; pasra@hotmail.com; 5 Department of Biochemistry, Phramongkutklao College of Medicine, Bangkok 10400, Thailand; montalee.th@pcm.ac.th

**Keywords:** venous thromboembolism (VTE), single-nucleotide polymorphism (SNP), polygenic risk score (PRS), genetic predisposition, MassARRAY, Thai population

## Abstract

**Background:** Venous thromboembolism (VTE) is a multifactorial disorder influenced by both genetic and environmental factors, with substantial variability in susceptibility across populations. Data on VTE-associated genetic variants in Asian populations, including Thais, remain limited. To address this, we developed a 39-single-nucleotide polymorphism (SNP) genotyping panel using the MassARRAY platform and evaluated its association with VTE in a Thai cohort. **Methods:** A total of 209 individuals, comprising 122 patients with objectively confirmed VTE and 87 age- and sex-matched healthy controls, were genotyped. Allele frequencies were compared, and associations with VTE were assessed. **Results:** Seven SNPs demonstrated significant associations: five risk alleles (*PROC* rs146922325, *ABO* rs8176743, *FGG* rs2066865, *F11* rs4253417, and *HIVEP1* rs169713) and two protective alleles (*F5* rs4524 and *TGFB2* rs57615042). To examine cumulative effects, a polygenic risk score (PRS) integrating genetic and clinical factors was constructed. Higher PRS was significantly associated with recurrence, particularly among patients with unprovoked VTE, conferring more than a threefold increase in recurrence risk (HR = 3.53, 95% CI: 1.04–10.2, *p* = 0.043). These findings provide the first systematic evidence of population-specific genetic risk factors for VTE in Thais and highlight the clinical potential of PRS for recurrence prediction. **Conclusions:** The MassARRAY-based panel offers a cost-effective, high-throughput strategy for simultaneous SNP detection, supporting scalable genomic studies and personalized risk stratification. Our results contribute to understanding the genetic architecture of VTE and highlight the value of incorporating non-European populations into genetic studies to advance precision medicine.

## 1. Introduction

Venous thromboembolism (VTE), encompassing deep vein thrombosis (DVT) and pulmonary embolism (PE), affects individuals across all ages, sexes, and ethnic backgrounds, and remains a significant cause of morbidity and mortality in various clinical settings [1]. In Western countries, the annual incidence of VTE is approximately 1–2 cases per 1000 individuals, resulting in substantial morbidity and mortality [2,3,4,5,6]. Although historically considered less prevalent in Asian populations, recent evidence indicates a rising incidence of VTE in this region [2,3]. In Thailand, however, national epidemiological data on VTE remain limited.

VTE is a multifactorial disorder influenced by both environmental and genetic factors [7,8,9,10], with genetic predisposition contributing substantially to overall susceptibility [11,12,13]. Numerous single-nucleotide polymorphisms (SNPs) in genes such as *F2*, *F5*, *FGG*, *PROC*, *PROS1*, *GP6*, *KNG1* and *SERPINC1* have been associated with inherited thrombophilia and increased VTE risk [9,10,14,15]. However, the allele frequencies and effect sizes of these variants differ considerably across populations, highlighting the importance of population-specific studies to better understand the genetic architecture of VTE and to enable more accurate risk stratification. Notably, limited data are available for Southeast Asian populations, including Thais.

While Sanger sequencing is considered the gold standard for genotyping, it is impractical for high-throughput applications involving multiple loci. The MassARRAY system, based on matrix-assisted laser desorption/ionization–time of flight (MALDI-TOF) mass spectrometry, has emerged as a cost-effective, high-throughput platform for simultaneous detection of multiple SNPs across numerous samples [16,17]. This technology has been successfully applied in various fields, including infectious disease genotyping [18], cancer susceptibility studies [19,20], and neurodevelopmental disorders [21], and is increasingly used to develop targeted gene panels for clinical research.

In this study, we aimed to develop a 39-SNP genetic screening panel, selected from previously published studies [9,15,22,23,24,25], using the MassARRAY platform to investigate genetic predisposition to VTE and evaluate their associations with VTE risk in a Thai population. We further constructed a polygenic risk score (PRS) and assessed its utility in predicting VTE recurrence and improving clinical risk stratification in this population.

## 2. Materials and Methods

### 2.1. Study Population

In this study, a total of 209 participants were consecutively enrolled, including 122 patients with VTE and 87 healthy controls. The 122 patients with symptomatic VTE presented to the Division of Hematology, Department of Medicine, Phramongkutklao Hospital, Bangkok, Thailand, between November 2024 and May 2025. All VTE patients were eligible for evaluation of genetic risk factors. Among them, 97 were diagnosed with DVT, 16 with PE, and 9 with both DVT and PE. The 87 healthy controls were recruited during the same period from individuals participating in an annual health check-up program at the same institution.

Inclusion criteria for all participants were as follows: Thai adults aged ≥18 years at the time of enrollment who provided written informed consent for participation, genetic testing, and data sharing, and from whom DNA samples of acceptable quality were obtained. For VTE patients, DVT was confirmed by Doppler ultrasonography or venography, whereas PE was diagnosed using ventilation–perfusion (V/Q) scans, computed tomography (CT), or pulmonary angiography. Patients with a history of blood component transfusion within the preceding 3 months, prior hematopoietic stem cell transplantation that could confound genotyping, or inability to provide documentation confirming a VTE diagnosis were excluded. For the control group, all participants were healthy individuals who demonstrated normal laboratory findings, including complete blood count, coagulation studies (prothrombin time, activated partial thromboplastin time, fibrinogen level, and D-dimer), lipid profile, and liver function tests. Controls were matched to cases by age and sex. Exclusion criteria for the control group included: a history of objectively confirmed venous thromboembolism (VTE); active malignancy or ongoing cancer treatment; current or long-term use of anticoagulant or antiplatelet therapy for any indication (e.g., atrial fibrillation, mechanical heart valve); known diagnosis of major inherited thrombophilia (e.g., protein C or protein S deficiency); pregnancy at the time of enrollment; use of oral contraceptives or hormone replacement therapy; major surgery or prolonged immobilization within the previous month; and a known family history of VTE.

Demographic data, including age, sex, and body mass index (BMI), were recorded for all participants. For VTE patients, clinical risk factors and baseline laboratory parameters—including coagulogram results, protein C activity, protein S activity, antithrombin activity, and antiphospholipid profiles—were retrospectively obtained from medical records. Medical data were accessed in June 2025 for research purposes. Identifiable participant information (e.g., hospital identification numbers) was accessible during data collection but was de-identified prior to analysis to maintain confidentiality.

The study was conducted in accordance with the Declaration of Helsinki. The study protocol was approved by the Committee of the Institutional Review Board, Royal Thai Army Medical Department (approval No. IRBRTA 1458/2567, project No. S060h/67, Approved date: 13 November 2024). Written informed consent was obtained from all patients and controls.

### 2.2. Blood Sample and DNA Preparation

Peripheral blood samples were collected in 2.0 mL EDTA tubes (Vacutainer, Greiner Bio-One, Stonehouse, UK). Genomic DNA was extracted using the FavorPrep™ Blood Genomic DNA Extraction Kit (Favorgen Biotech Corp., Ping-Tung, Taiwan), according to the manufacturer’s instructions. The DNA samples were aliquoted and stored at −20 °C until use.

### 2.3. Candidate Gene and SNP Selection

In this study, VTE-associated SNPs were selected based on a comprehensive review of the literature [9,15,22,23,24,25], as well as their relevance to the pathophysiology of venous thrombosis. A total of 39 SNPs from 26 genes were included, comprising 30 well-established and 9 novel VTE-associated loci. All 30 well-established loci, including *PROC* rs146922325, *THBD* rs16984852, *F2* rs1799963, rs3136516 and rs191945074, *F5* rs4524, *ABO* rs687289, rs2519093, rs8176719, rs8176743, rs8176749 and rs9411377, *FGG* rs2066864 and rs2066865, *PAI-1* rs2227631, *APOH* rs8178847, *NOS3* rs1799983, *F11* rs2036914, rs2289252 and rs4253417, *MTHFR* rs1801133, *SERPINC1* rs2227589, *HIVEP1* rs169713, *TSPAN15* rs78707713, *KNG1* rs710446, *PROS1* rs6795524, *CYP4V2* rs13146272, *VWF* rs1063856 and *GP6* rs1613662, showed statistically significant association with VTE (*p* < 0.05, or *p* < 5 × 10^−8^ for genome-wide association studies [GWAS]) and reported odds ratio (OR) >1.10 or <0.90.

In addition, 9 novel VTE-associated loci were selected, based on the study by Ghouse et al. [13]. These included *SERPINC1* rs2227624, *PDIA3* rs139974673, *PPP1R3B* rs34290760, *SYK* rs10993706, *TGFB2* rs57615042, *MTOR* rs12097293, *KNG1* rs5030062, *ZFPM1* rs55823018, and *XXYLT1* rs56324901. Each variant met the predefined selection criteria of a genome-wide significance threshold (*p* < 5 × 10^−8^) and an effect allele frequency (EAF) greater than 0.1.

### 2.4. Multiplex Polymerase Chain Reaction

All targeted SNPs were inputted into AgenaCx design platform (Agena Bioscience, Inc., San Diego, CA, USA), based on the GRCh38/hg38 reference genome (Available from: https://agenacx.com/, accessed on 15 June 2025) for primer and assay design, following the manufacturer’s recommendations. The primer sequences used for the assays are provided in Supplementary Tables S1 and S2.

Multiplex polymerase chain reaction (PCR) amplification was performed in a total reaction volume of 5.0 μL, containing 500 nM of primer mix (forward and reverse primers), 2.0 mM MgCl_2_, 500 μM dNTPs, 1× PCR buffer, 0.2 U/μL DNA polymerase, and 10–20 ng/μL of genomic DNA template. Cycling was carried out on a SimpliAmp™ Thermal Cycler (Thermo Fisher Scientific, Waltham, MA, USA) under the following conditions: initial denaturation at 95 °C for 2 min (1 cycle); 48 cycles of denaturation at 95 °C for 30 s, annealing at 56 °C for 30 s, and extension at 72 °C for 1 min; followed by a final extension at 72 °C for 5 min.

Residual dNTPs from the previous step were removed by shrimp alkaline phosphatase (SAP) treatment. The reaction was carried out in a total volume of 2.0 μL, containing 0.17 μL of 10× SAP buffer, 1.53 μL of high-performance liquid chromatography (HPLC)-grade distilled water (DW), and 0.3 μL of SAP enzyme. Reaction tubes were incubated at 37 °C for 40 min, followed by enzyme inactivation at 85 °C for 5 min.

The final step, the single-base extension (SBE) reaction, was performed by adding 2.0 μL of the extension reaction cocktail—comprising 0.6 μL of HPLC-grade DW, 0.2 μL of iPLEX^®^ Pro buffer, 0.2 μL of iPLEX^®^ Pro terminator mix, 0.05 μL of iPLEX^®^ Pro enzyme, and 0.95 μL of extension primers—into the PCR products from the previous step. The PCR cycling conditions were as follows: initial denaturation at 94 °C for 30 s (1 cycle), followed by 48 cycles of denaturation at 94 °C for 5 s, annealing at 55 °C for 5 s, and extension at 80 °C for 5 s; with a final extension at 72 °C for 3 min. A PCR internal-control, *GAPDH* gene, was added in every reaction to check the quality of the PCR.

### 2.5. SNPs Genotyping Using MassARRAY

Genotyping was performed using the Agena MassARRAY platform (Agena Bioscience, CA, USA). Briefly, the SBE products were desalted using SpectroCLEAN resin and dispensed onto a SpectroCHIP using a nano-dispenser. The chips were then loaded into a matrix-assisted laser desorption/ionization time-of-flight mass spectrometer (MALDI-TOF MS) for molecular mass analysis of the SBE products. The resulting mass spectra and base-calling data were analyzed and interpreted using MassARRAY^®^ Typer Viewer v4.0 software (Agena Bioscience, CA, USA). All SNPs passed standard quality control filters with call rates > 95% and no significant deviation from Hardy–Weinberg equilibrium in controls. To assess assay reproducibility, 20 samples (10% of the total) were randomly selected and reanalyzed in independent runs. The results showed 100% concordant genotypes, indicating excellent inter-assay reproducibility. In addition, genotyping accuracy was validated by Sanger sequencing of randomly selected samples (*n* = 5) using the same primer set for each locus, which showed complete concordance with the MassARRAY results.

### 2.6. Polygenic Risk Score Calculation

A PRS was calculated to evaluate the cumulative effect of multiple genetic variants on VTE susceptibility and to develop a predictive model for VTE recurrence. The PRS for each individual was computed based on the profile of 39 SNPs included in the panel. The PRS was calculated using the following formula:

PRS = β_1_ × SNP_1_ + β_2_ × SNP_2_ + … + β_n_ × SNP_n_,

where β represents the natural logarithm of the OR (log[OR]) for each SNP, and SNP refers to the number of risk alleles (0, 1, or 2) carried by the individual.

The resulting PRS values were then standardized using the Z-score transformation:

Standardized PRS = (PRS−mean PRS of controls)/standard deviation (SD) of PRS in controls.

### 2.7. Statistical Analysis

Continuous variables were presented as mean ± SD, median, and interquartile range (IQR), and were compared using Student’s *t*-test or Mann–Whitney U test, as appropriate. Categorical variables were summarized as frequencies and percentages. Allele frequencies (AFs) of each SNP were compared between cases and controls using the Chi-square (*χ*^2^) test or Fisher’s exact test, depending on the expected cell counts. Univariate and multivariate logistic regression analyses were conducted to estimate the effect sizes, expressed as ORs with 95% confidence intervals (CIs). To assess the statistical power of the observed effect sizes, we conducted a retrospective power analysis using the Genetic Association Study Power Calculator (Available from: https://csg.sph.umich.edu/abecasis/cats/gas_power_calculator/, accessed on 15 June 2025). Assuming a log-additive model, α = 0.05, and varying EAFs, we estimated the detectable ORs at 80% power for our case–control cohort. Kaplan–Meier analysis was used to assess the association between standardized PRS and 2-year incidence of recurrent VTE. Hazard ratios (HRs) with 95% CIs were calculated using Cox proportional hazards regression.

All statistical analyses were performed using IBM SPSS Statistics for Windows, Version 20.0 (IBM Corp., Armonk, NY, USA), and GraphPad Prism, Version 9 (GraphPad Software, San Diego, CA, USA). A *p*-value of less than 0.05 was considered statistically significant.

## 3. Results

### 3.1. Baseline Clinical Characteristics of 122 VTE Patients

The baseline clinical characteristics of the 122 patients with VTE and the 87 healthy controls included in this study are summarized in Table 1. The median age was 44.5 years (IQR, 32–57 years). Although VTE incidence increases significantly in individuals older than 60 years and this age group is considered high-risk [2,3,8], the majority of patients in our study (77.9%) were diagnosed with VTE before the age of 60. Among VTE patients, 54.9% were male and 45.1% were female. The mean BMI was 24.5 ± 4.0 kg/m^2^, with 10.7% classified as overweight. There were no significant differences between cases and controls in terms of age, sex, or BMI (all *p* > 0.05, Table 1).

Among the VTE cases, 79.5% were diagnosed with DVT, 13.1% with PE alone, and 7.4% with both DVT and PE. Based on clinical risk classification, 70.5% of patients had provoked VTE, while 29.5% were classified as unprovoked cases (Table 1).

The most common risk factors identified in this cohort were the presence of antiphospholipid antibodies, underlying malignancy, and a recent history of trauma or surgery (within one month). Deficiencies in natural anticoagulants were also observed, with protein C, protein S, and antithrombin deficiencies found in 6.6%, 5.7%, and 0.8% of patients, respectively. During the two-year follow-up period, 49.2% of patients experienced recurrent VTE (Table 1).

### 3.2. Allele Frequencies of the 39 VTE Susceptibility SNPs Among Thai Patients and Controls

**Figure 1:**
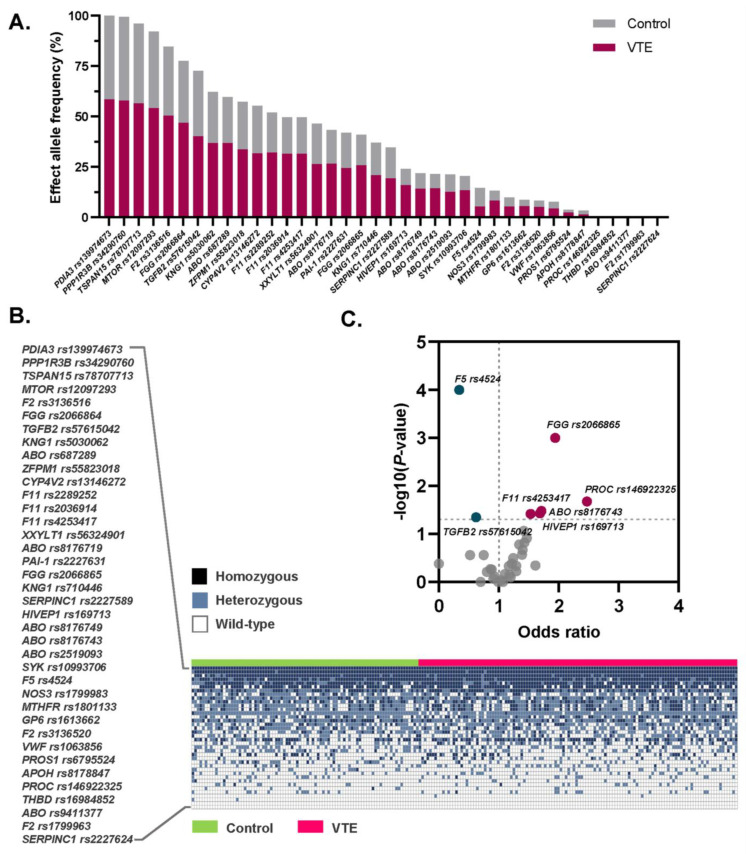
Frequency and distribution of the 39 VTE-associated SNPs among the Thai population. (**A**) Bar chart illustrating the risk allele frequencies of each SNP in Thai patients with VTE compared to healthy controls. (**B**) Heatmap showing the genotype distribution (wild-type, heterozygous and homozygous risk alleles) for each SNP in both VTE patients and controls. (**C**) Volcano plot highlighting statistically significant SNPs associated with VTE risk: five risk variants (in red) and two protective variants (in green).

The allele frequencies and genotype distributions of 39 SNPs previously associated with VTE were analyzed and are presented in Figure 1. Risk allele frequencies for each SNP in Thai VTE patients compared to healthy controls are shown in Figure 1A, while genotype distributions (wild-type, heterozygous, and homozygous risk alleles) are illustrated in a heatmap in Figure 1B.

### 3.3. Association Between the Selected SNPs and Risk of VTE

The effect allele frequencies and their associations with VTE risk among Thai patients and healthy controls are summarized in Table 2. All observed genotypic distributions conformed to Hardy–Weinberg equilibrium expectations. Univariate logistic regression identified seven SNPs significantly associated with VTE risk (Table 2 and Figure 1C), including five risk alleles: *PROC* rs146922325 (OR = 1.94, 95% CI: 1.08–3.60), *ABO* rs8176743 (OR = 1.71, 95% CI: 1.07–2.80), *FGG* rs2066865 (OR = 1.94, 95% CI: 1.30–2.90), *F11* rs4253417 (OR = 1.53, 95% CI: 1.02–2.20), and *HIVEP1* rs169713 (OR = 1.69, 95% CI: 1.05–2.66), as well as two protective variants: *F5* rs4524 (OR = 0.34, 95% CI: 0.19–0.60) and *TGFB2* rs57615042 (OR = 0.62, 95% CI: 0.40–0.98). These findings remained statistically significant after adjustment for potential confounders including age, sex, and BMI. The adjusted ORs were 1.70 (95% CI: 1.05–2.79) for *PROC* rs146922325; 1.60 (95% CI: 1.01–2.55) for *ABO* rs8176743; 1.77 (95% CI: 1.18–2.66) for *FGG* rs2066865; 1.58 (95% CI: 1.07–2.35) for *F11* rs4253417; and 1.61 (95% CI: 1.02–2.53) for *HIVEP1* rs169713. In contrast, persistent protective effects were observed for *F5* rs4524 and *TGFB2* rs57615042, with adjusted ORs of 0.42 (95% CI: 0.24–0.72) and 0.56 (95% CI: 0.35–0.90), respectively.

All SNPs that showed statistically significant associations with VTE in our analysis demonstrated high statistical power (>80%) to detect their reported effects. In contrast, variants such as *APOH* rs8178847, *F2* rs1799963 and *PROS1* rs6795524, which exhibited low allele frequencies and modest literature-based effect sizes, were underpowered (estimated power < 50%). These findings support the reliability of associations observed for well-powered SNPs and highlight the importance of larger sample sizes or meta-analytic approaches to accurately assess the effects of low-frequency variants.

### 3.4. Genotype-Phenotype Associations Based on Genetic Models

**Figure 2:**
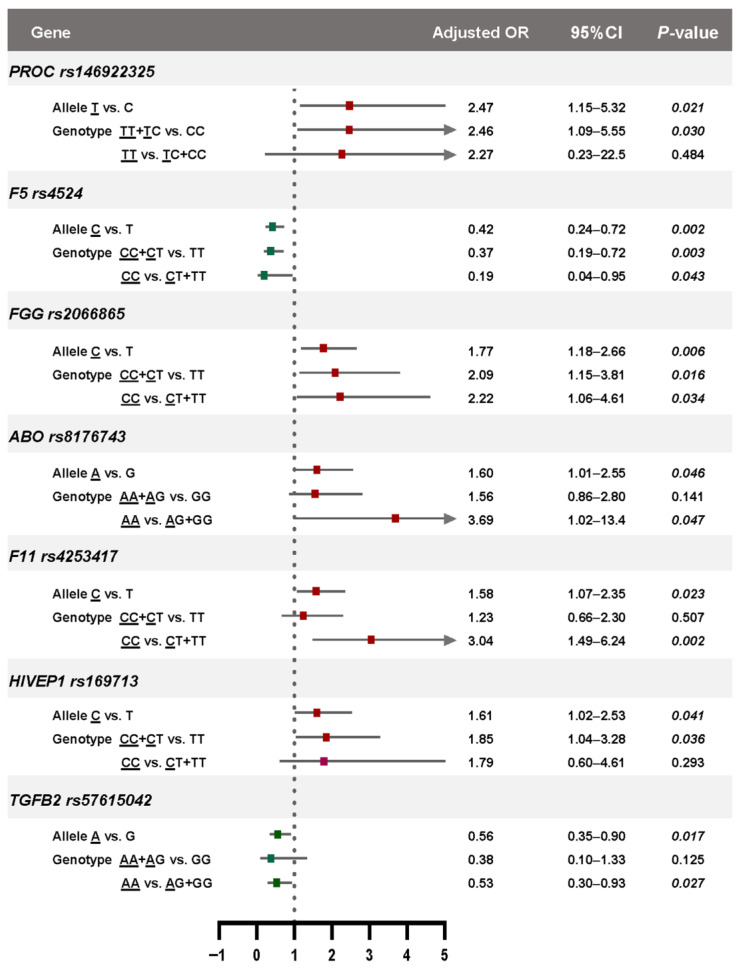
Allelic and genetic risk models of the seven SNPs significantly associated with VTE risk in the Thai population. Red squares indicate risk effects, and green squares indicate protective effects. Adjusted odds ratios and 95% confidence intervals were calculated after adjusting for age, sex, and BMI. Risk alleles are indicated by underlined letters, and statistically significant *p*-values are presented in italics.

Genotyping models were further analyzed to investigate the genotype–phenotype relationships of the significant SNPs (Figure 2). *PROC* rs146922325 and *HIVEP1* rs169713 were significantly associated with an increased risk of VTE under the dominant model, whereas *ABO* rs8176743, *F11* rs4253417, and *TGFB2* rs57615042 showed significant associations under the recessive model. Interestingly, *FGG* rs2066865 was significantly associated with VTE risk under both dominant and recessive models, suggesting that both heterozygous and homozygous carriers of the risk allele may have increased susceptibility. The stronger association observed under the recessive model (adjusted ORs: 2.02 vs. 2.22) may reflect an additive or dose-dependent effect of the risk allele. In contrast, *F5* rs4524 was associated with a reduced risk of VTE under both dominant and recessive models, with a stronger protective effect observed under the recessive model (adjusted ORs: 0.37 vs. 0.19). These findings suggest that this variant may confer a strong protective effect, particularly in individuals homozygous for the protective allele.

### 3.5. Polygenic Risk Score and Risk Prediction of VTE

**Figure 3:**
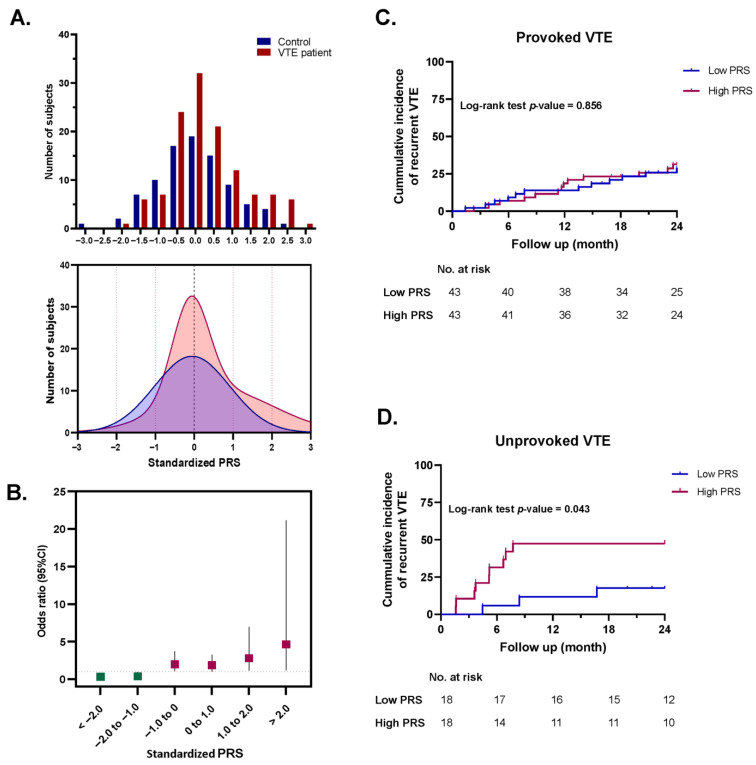
Population-specific polygenic risk score (PRS) model and its clinical application in predicting VTE recurrence in Thai patients. (**A**) Distribution of standardized PRS among VTE patients and healthy controls. (**B**) Odds ratios with 95% confidence intervals for VTE risk stratified by standardized PRS categories. Red squares indicate risk effects, and green squares indicate protective effects. Kaplan–Meier plots illustrating the cumulative incidence of recurrent thrombotic events in Thai patients with (**C**) provoked (*n* = 86) and (**D**) unprovoked (*n* = 36) VTE, stratified by high vs. low PRS.

The distribution of standardized PRS among Thai VTE patients and healthy controls is shown in Figure 3A. The mean ± SD of the standardized PRS in VTE patients and controls was 6.92 ± 6.64 and 9.54 ± 10.1, respectively. Logistic regression analysis showed that increasing standardized PRS values were significantly associated with higher OR of VTE, as illustrated in Figure 3B. Notably, individuals with a standardized PRS greater than −1.0 exhibited a significantly increased risk of thrombosis.

### 3.6. Association Between the Standardized PRS and Risk of Recurrent VTE

In this study, VTE patients were stratified into two subgroups based on their underlying risk classification: provoked VTE (*n* = 86) and unprovoked VTE (*n* = 36). Within each subgroup, patients were further categorized into low-PRS and high-PRS groups according to the median standardized PRS of the overall cohort. The 2-year incidence of recurrent VTE was assessed by retrospective review of medical records. Time-to-event was calculated from the date of anticoagulant discontinuation to the date of confirmed recurrent VTE. All recurrent events were objectively confirmed using standard imaging modalities, consistent with the methods used for the initial diagnosis

Among patients with provoked VTE, there was no significant difference in the incidence of recurrent VTE between the low-PRS and high-PRS groups (log-rank *p* = 0.856, Figure 3C). Interestingly, unprovoked VTE patients with a high standardized PRS exhibited a significantly higher recurrence rate compared to those with low PRS (log-rank *p* = 0.043, HR = 3.53, 95% CI: 1.04–10.2, Figure 3D).

## 4. Discussion

In this study, we developed a primary genetic screening panel comprising 39 VTE-associated SNPs using the MassARRAY platform to explore genetic predisposition to VTE among Thai patients. We also conducted a comprehensive analysis integrating both genetic and clinical variables and applied standardized PRS modeling to evaluate its utility in predicting VTE recurrence. Our findings provide novel insights into the genetic variations associated with VTE in the Thai population and highlight the potential of integrating genomic and clinical data for enhanced risk stratification, particularly in individuals with unprovoked VTE. To the best of our knowledge, this is the first study to develop and implement a VTE-specific SNP panel alongside PRS modeling in a Thai cohort, offering a region-specific approach to thrombosis risk assessment.

During the screening panel development phase, the 39 SNPs selected for this study were based on robust evidence from prior GWAS, candidate gene studies and meta-analyses that demonstrated associations with VTE or related thrombotic pathways. These variants span genes involved in key hemostatic processes, including coagulation (e.g., *F5*, *F2* and *F11*), anticoagulation (*SERPINC1*, *PROS1*), fibrinolysis (*FGG*), platelet activation (*GP6*) and vascular function (*ABO*, *VWF*). Others, including *TSPAN15* and *SLC44A2*, were identified through large multi-ethnic meta-analyses and may contribute to thrombotic risk via less well-understood mechanisms [25]. Although the well-established genetic risk locus factor V Leiden (*FVL*; rs6025) is known to be strongly associated with VTE in Western populations [26,27], this variant was not included in our genotyping panel due to its extremely low prevalence in the Thai population [28,29]. However, we screened all enrolled patients for this variant using allele-specific PCR and identified only one individual with the heterozygous *FVL* genotype, further supporting the limited relevance of this variant in the pathogenesis of VTE among Thais.

Our results revealed that the top five SNPs, including *PDIA3* rs139974673, *PPP1R3B* rs34290760, *TSPAN15* rs78707713, *MTOR* rs12097293, and *F2* rs3136516, exhibited the highest allele frequencies. *PDIA3* rs139974673, *PPP1R3B* rs34290760 and *MTOR* rs12097293 were initially identified as novel VTE-associated loci by Ghouse et al. [13], with reported effect allele frequencies of 0.98, 0.93, and 0.77, respectively. In our cohort, the allele frequencies for these SNPs were 1.00, 0.99, and 0.92, respectively. The risk allele frequency of *TSPAN15* rs78707713 has been reported to range from 0.76 to 0.77 in global populations [25], whereas we observed a higher frequency of 0.94 in the Thai population, consistent with the elevated frequency reported in African Americans [30]. The G allele of *F2* rs3136516 was observed at a frequency of 0.84 in our cohort, consistent with frequencies reported in populations of African descent (0.82–0.91) [31]. By contrast, the frequency is lower in Caucasians (0.44–0.50) [31,32]. Despite their high prevalence, none of these variants were significantly associated with VTE risk in our population, indicating potential population-specific effects or non-pathogenic roles of these alleles. However, further studies are needed to elucidate the functional roles of these variants in VTE pathogenesis.

In contrast, *F2* rs1799963 (prothrombin G20210A) and *SERPINC1* rs2227624 (antithrombin Dublin variant) were not detected in the Thai population. Prothrombin G20210A has previously been reported as a rare variant among Thais [28,29]. While specific data on the antithrombin Dublin variant in Thailand are unavailable, its prevalence is presumed to be extremely low among East Asian populations based on the ClinVar database [33].

Genetic profile analysis of 122 VTE patients and 87 healthy controls identified seven SNPs significantly associated with VTE risk, including five risk alleles: *PROC* rs146922325, *ABO* rs8176743, *FGG* rs2066865, *F11* rs4253417, and *HIVEP1* rs169713, and two protective variants: *F5* rs4524 and *TGFB2* rs57615042. Notably, these associations remained significant after adjustment for age, sex, and BMI, highlighting their potential as independent genetic markers for VTE susceptibility in the Thai population.

The *PROC* rs146922325 variant (c.565C>T; p.Arg189Trp), also historically referred to as R147W or R189W, has been extensively studied, particularly in Asian populations, due to its strong association with hereditary protein C deficiency and increased thrombotic risk [24,34,35,36]. Notably, allele frequencies among VTE patients have been reported at 0.108 in Singapore, 0.105 in China, and 0.227 in Malaysia [36]. In our Thai cohort, the observed frequency was 0.164, providing additional evidence for the enrichment of this variant in Asian populations. This frequency is notably higher than that previously reported in the general Thai population (0.03) and in thrombotic Thai children (0.075) [37]. Moreover, a recent study demonstrated that this variant results in approximately a 30% reduction in protein C secretion in Thai patients [38], highlighting its potential role in the genetic predisposition to VTE within this population.

The *ABO* rs8176743 variant is a missense mutation located in exon 7 that affects the glycosyltransferase enzyme responsible for determining A, B, and AB blood groups [39]. The rs8176743-T allele, linked to the B blood group phenotype, has been associated with an increased risk of VTE [39]. This allele is moderately common in European populations, with a reported frequency of approximately 0.10 to 0.15, and is well-documented in global genomic datasets [39]. The *FGG* rs2066865 variant is an intronic polymorphism that may influence alternative splicing or regulate fibrinogen levels, potentially contributing to a prothrombotic state. The T risk allele has been reported at a frequency of 0.283 in Caucasian populations [40].

Although both *F11* rs4253417 and *HIVEP1* rs169713 variants have previously been identified as susceptibility loci for VTE [24,41,42,43], studies exploring their functional roles and population-specific allele distributions remain limited. Although the exact functional consequences of *F11* rs4253417 and *HIVEP1* rs169713 remain unclear, *F11* encodes coagulation factor XI, suggesting that variants in this gene may influence plasma FXI levels and modulate thrombosis risk, as previously identified in patients with ischemic stroke [42]. *HIVEP1*, a transcription factor implicated in immune gene regulation [44], warrants further mechanistic studies to elucidate its potential role in VTE.

Notably, in our cohort, all of these significant risk alleles were observed at higher frequencies among Thai patients with VTE compared to controls, indicating a potential population-specific enrichment. These findings support their potential contributory role in thrombotic risk, particularly in the Thai or Southeast Asian populations. To validate these associations, further functional studies and comprehensive genomic analyses in larger, ethnically diverse cohorts are warranted. These investigations potentially provide insights into the biological mechanisms underlying VTE susceptibility and support the development of more accurate, population-specific risk prediction tools.

The two protective variants, *F5* rs4524 and *TGFB2* rs57615042, were observed at lower allele frequencies in Thai VTE patients compared to controls (0.09 vs. 0.22 and 0.69 vs. 0.78, respectively). The *F5* rs4524 variant (R534Q) is known to modulate factor V cofactor activity and has been linked to a decreased risk of thrombosis in individuals without the FVL mutation [45]. Although less extensively studied in the context of VTE, the *TGFB2* rs57615042 variant may influence endothelial repair mechanisms or inflammatory responses, given the established role of TGFB2 in vascular homeostasis and immune modulation [46]. The protective associations observed in our study, particularly the higher allele frequencies among healthy Thai controls, imply a possible population-specific effect. Nonetheless, due to limited data on this variant in Asian populations, further research is required.

Genotype–phenotype association analysis further refined these findings by applying both dominant and recessive genetic models. The *FGG* rs2066865 variant was significantly associated with VTE under both inheritance patterns, with a stronger effect observed in the recessive model, suggesting a possible additive or dose-dependent effect. In contrast, the protective *F5* rs4524 variant showed a strong inverse association with VTE, particularly under the recessive model, further supporting its potential protective role when present in homozygous form.

Importantly, this study assessed the utility of a standardized PRS derived from all 39 SNPs. A higher PRS was significantly associated with an increased risk of VTE and remained predictive in recurrence models. Notably, the standardized PRS may serve as a valuable prognostic biomarker for identifying individuals with unprovoked VTE at elevated risk of recurrence, thereby supporting personalized long-term management and guiding decisions regarding secondary prevention strategies in this high-risk subgroup. However, despite the strong associations between the PRS and recurrence, its clinical application depends on prospective validation and cost–benefit analyses in real-world settings. This preliminary evaluation also identified several variants with undetected or negligible prevalence among the Thai population. In future work, this genetic screening panel could be refined and expanded to enhance predictive accuracy and better capture population-specific risk profiles, ultimately improving its clinical utility in the Thai population.

Several limitations of our study should be acknowledged. First, although the sample size is comparable to previous genetic studies in Southeast Asian populations, it may limit the ability to detect modest effect sizes or associations with rare variants. Second, because all enrolled patients were selected based on eligibility for investigation of genetic risk factors, the median age of our cohort was relatively low, and the observed incidence of recurrent VTE was higher than reported in several other populations [47,48,49]. Future studies with a broader range of patients across different age groups and clinical backgrounds, ideally through multicenter or population-based recruitment, are needed to mitigate this limitation. Third, our focus on a targeted SNP panel, while enabling efficient genotyping and interpretation, may have limited the ability to capture all relevant genetic determinants of VTE. Finally, the retrospective nature of clinical data collection introduces the potential for missing or incomplete records, particularly regarding recurrence events.

## 5. Conclusions

In conclusion, the MassARRAY-based panel provides a cost-effective, high-throughput approach for simultaneous SNP detection, enabling scalable genomic studies and personalized risk stratification. Our findings underscore the importance of integrating genetic data with clinical variables for individualized risk assessment and management of VTE. A standardized PRS may serve as a valuable prognostic biomarker for patients with unprovoked VTE at high risk of recurrence, supporting personalized management and guiding secondary prevention. Further functional studies and larger prospective cohorts are warranted to validate these findings and facilitate the implementation of population-specific risk prediction models in clinical practice.

## Figures and Tables

**Table 1:** Clinical characteristics of 122 Thai patients with VTE and 87 healthy controls included in this study.

Characteristics		VTE Patients (*n* = 122)	Healthy Controls (*n* = 87)
Age, year	Median (IQR)	44.5 (32–57)	45.0 (36–56)
	Age < 60 yr, *n* (%)	95 (77.9)	72 (82.8)
	Age ≥ 60 yr, *n* (%)	27 (22.1)	15 (17.2)
BMI, kg/m^2^	Mean ± SD	24.5 ± 4.0	24.9 ± 4.4
	BMI < 30.0 kg/m^2^, *n* (%)	109 (89.3)	77 (88.5)
	BMI ≥ 30.0 kg/m^2^, *n* (%)	13 (10.7)	10 (11.5)
Sex, *n* (%)			
	Male	67 (54.9)	51 (58.6)
	Female	55 (45.1)	36 (41.4)
Diagnosis, *n* (%)			
	DVT	97 (79.5)	N/A
	PE	16 (13.1)	N/A
	PE + DVT	9 (7.4)	N/A
Risk group, *n* (%)			
	Provoked VTE	86 (70.5)	N/A
	Unprovoked VTE	36 (29.5)	N/A
VTE risk factors, *n* (%)			
	Antiphospholipid syndrome	17 (13.9)	0 (0.0)
	Active cancer	11 (9.0)	0 (0.0)
	Recent trauma or surgery (≤1 month)	9 (7.4)	0 (0.0)
	Protein C deficiency	8 (6.6)	0 (0.0)
	Protein S deficiency	7 (5.7)	0 (0.0)
	Oral contraceptive used	7 (5.7)	0 (0.0)
	Immobility	3 (2.5)	0 (0.0)
	Antithrombin deficiency	1 (0.8)	0 (0.0)
	Ongoing hormone therapy	1 (0.8)	0 (0.0)
Recurrent VTE ^a^, *n* (%)			
	Recurrence	60 (49.2)	N/A
	No recurrence	62 (50.8)	N/A

^a^ Recurrent VTE was defined as the occurrence of a thrombotic event within two years of stopping anticoagulant treatment during follow-up. Abbreviations: BMI, body mass index; DVT, deep vein thrombosis; IQR, inter-quartile ranges; N/A, not applicable; PE, pulmonary embolism; SD, standard deviation; VTE, venous thromboembolism.

**Table 2:** Distribution frequencies of 39 VTE-associated SNPs and their associations with VTE risk in the Thai population.

Gene	rs ID	Alt/Ref	Literature OR [9,13,15,22,23,24,25]	EAF Cases/Controls	Univariate Analysis		Multivariate Analysis	
					OR (95% CI)	*p*-Value	Adjusted OR ^a^ (95%CI)	*p*-Value
*PROC*	rs146922325	**T**/C	6.91	0.16/0.09	1.94 (1.08–3.60)	0.041	1.71 (1.05–2.79)	0.032
*THBD*	rs16984852	**A**/C	2.80	0.01/0.00	Infinity (0.33–infinity)	0.513	-	-
*F2*	rs1799963	**A**/G	1.88	0.00/0.00	Infinity (0.00–infinity)	>0.999	-	-
*F2*	rs191945074	**T**/C	1.86	0.09/0.08	1.07 (0.54–2.21)	>0.999	-	-
*ABO*	rs8176719	**C**/-	1.85	0.46/0.40	1.24 (0.83–1.83)	0.317	-	-
*ABO*	rs8176743	**T**/C	1.76	0.26/0.17	1.71 (1.07–2.80)	0.033	1.60 (1.01–2.55)	0.046
*FGG*	rs2066865	**A**/G	1.56	0.52/0.36	1.94 (1.30–2.90)	0.001	1.77 (1.18–2.66)	0.006
*PAI-1*	rs2227631	**A**/G	1.55	0.42/0.42	0.99 (0.66–1.46)	>0.999	-	-
*APOH*	rs8178847	**T**/C	1.55	0.02/0.05	0.52 (0.18–1.56)	0.275	-	-
*NOS3*	rs1799983	**T**/G	1.41	0.14/0.14	1.29 (0.71–2.32)	0.464	-	-
*ABO*	rs2519093	**T**/C	1.40	0.24/0.21	1.22 (0.76–1.98)	0.411	-	-
*ABO*	rs9411377	**A**/C	1.36	0.00/0.00	0.00 (0.00–6.42)	0.416	-	-
*ABO*	rs687289	**A**/G	1.34	0.63/0.55	1.42 (0.97–2.10)	0.086	-	-
*F11*	rs2036914	**T**/C	1.32	0.19/0.24	0.75 (0.47–1.20)	0.275	-	-
*MTHFR*	rs1801133	**T**/C	1.30	0.09/0.11	0.80 (0.42–1.51)	0.617	-	-
*F11*	rs2289252	**T**/C	1.26	0.55/0.48	1.34 (0.90–1.99)	0.165	-	-
*ABO*	rs8176749	**T**/C	1.23	0.24/0.18	1.43 (0.89–2.31)	0.151	-	-
*F11*	rs4253417	**C**/T	1.22	0.54/0.43	1.53 (1.02–2.20)	0.038	1.58 (1.07–2.35)	0.023
*SERPINC1*	rs2227589	**T**/C	1.20	0.33/0.37	1.17 (0.77–1.76)	0.467	-	-
*HIVEP1*	rs169713	**C**/T	1.20	0.28/0.19	1.69 (1.05–2.66)	0.037	1.61 (1.02–2.53)	0.041
*TSPAN15*	rs78707713	**T**/C	1.20	0.97/0.95	1.61 (0.66–4.18)	0.452	-	-
*FGG*	rs2066864	**A**/G	1.20	0.80/0.74	1.47 (0.94–2.30)	0.122	-	-
*KNG1*	rs710446	**G**/A	1.19	0.36/0.39	0.86 (0.58–1.29)	0.537	-	-
*F2*	rs3136516	**G**/A	1.19	0.86/0.82	1.39 (0.82–2.34)	0.270	-	-
*PROS1*	rs6795524	**G**/A	1.19	0.04/0.03	1.20 (0.44–3.31)	0.801	-	-
*CYP4V2*	rs13146272	**A**/C	1.17	0.54/0.56	0.93 (0.64–1.38)	0.765	-	-
*VWF*	rs1063856	**C**/T	1.16	0.07/0.08	0.91 (0.45–1.93)	0.853	-	-
*GP6*	rs1613662	**G**/A	1.15	0.09/0.07	1.29 (0.65–2.62)	0.596	-	-
*F5*	rs4524	**C**/T	0.88	0.09/0.22	0.34 (0.19–0.60)	< 0.001	0.42 (0.24–0.72)	0.002
*SERPINC1*	rs2227624	**A**/T	0.99	0.00/0.00	Infinity (0.00–infinity)	> 0.999	-	-
*PDIA3*	rs139974673	**T**/C	0.98	1.00/1.00	Infinity (0.00–infinity)	> 0.999	-	-
*PPP1R3B*	rs34290760	**C**/G	0.96	0.99/0.99	0.70 (0.05–6.06)	> 0.999	-	-
*SYK*	rs10993706	**A**/G	0.93	0.23/0.17	1.40 (0.85–2.34)	0.215	-	-
*TGFB2*	rs57615042	**A**/G	0.89	0.69/0.78	0.62 (0.40–0.98)	0.045	0.56 (0.35–0.90)	0.017
*MTOR*	rs12097293	**A**/G	0.77	0.93/0.91	1.18 (0.58–2.48)	0.714	-	-
*KNG1*	rs5030062	**A**/C	0.63	0.63/0.61	1.10 (0.74–1.63)	0.683	-	-
*ZFPM1*	rs55823018	**T**/C	0.31	0.58/0.57	1.04 (0.71–1.55)	0.920	-	-
*XXYLT1*	rs56324901	**A**/G	0.20	0.45/0.48	0.88 (0.59–1.31)	0.551	-	-

^a^ Adjusted odds ratios and 95% confidence intervals were calculated after adjusting for age, sex, and body mass index. Altered or effect alleles are indicated by underlined letters. Statistically significant effect sizes and corresponding *p*-values are highlighted in bold. Abbreviations: Alt/Ref, Altered/Reference alleles; CI, confidence interval; EAF, effect allele frequency; OR, odds ratio.

## Data Availability

The original contributions presented in this study are included in the article/Supplementary Material. Further inquiries can be directed to the corresponding author.

## Supplementary Materials

The following supporting information can be downloaded at: https://www.mdpi.com/article/10.3390/medsci13040282/s1. This study includes Supplementary Tables S1 and S2, which are provided in the Supporting Information file (DOCX format). Supplementary Table S1 Primer sequences for genotyping the 39 VTE-associated SNPs and GAPDH control. Supplementary Table S2 Extension primer sequences for the 39 VTE-associated SNPs and GAPDH control.

## Abbreviations

The following abbreviations are used in this manuscript:

VTE	Venous thromboembolism
DVT	Deep vein thrombosis
PE	Pulmonary embolism
SNPs	Single-nucleotide polymorphisms
MALDI-TOF	Matrix-assisted laser desorption/ionization–time of flight
PRS	Polygenic risk score
CT	Computed tomography
BMI	Body mass index
EAF	Effect allele frequency
PCR	Polymerase chain reaction
SAP	Shrimp alkaline phosphatase
HPLC	High-performance liquid chromatography
DW	Distilled water
OR	Odds ratio
SBE	Single-base extension
MS	Mass spectrometer
IQR	Interquartile range
AFs	Allele frequencies
HR	Hazard ratio
CI	Confidence interval
