# Supplementary material for: Development of a MassARRAY Genotyping Platform and Its Clinical Application for Venous Thromboembolism Risk Assessment in Thai Patients
Source: Med Sci (Basel). 2025 Nov 24;13(4):282. doi: 10.3390/medsci13040282 (PMC12735279; doi:10.3390/medsci13040282)
Supplement: Supplementary file 1 [file medsci-13-00282-s001.zip › medsci-3969664-supplementary.pdf]

Supporting Information file:

Genetic Risk Assessment of Venous Thromboembolism in Thai Patients: Development and Clinical Application of a 39-SNP MassARRAY Panel

Dollapak Apipongrat, Chonlada Laoruangroj, Oytip Nathalang, Pasra Arnutti, Montalee Theeraapisakkun, Wittawat Chantkran

Corresponding author:

Wittawat Chantkran,

Department of Pathology, Phramongkutklao College of Medicine, Her Royal Highness Princess Bejaratana Building, 317 Ratchawithi Road, Ratchathewi District, Bangkok 10400, Thailand. Email: chantkran@yahoo.com

Supplementary Table S1 Primer sequences for genotyping the 39 VTE-associated SNPs and GAPDH control

SNP_ID	Reverse primer	Forward primer	PCR product length (bp)
rs78707713	ACGTTGGATGGGAAGCTGAGCAGAAATGAG	ACGTTGGATGCTTTCGACTTCACCTTCCAC	122
rs56324901	ACGTTGGATGCCCAGTGTCTACAACTATAC	ACGTTGGATGGTTTGAGTGATCCTCCCAG	109
rs60509203	ACGTTGGATGGGAGGAATTCATAACTACC	ACGTTGGATGCTCACAAACACAATTTCCTC	136
rs12097293	ACGTTGGATGACTGGATTCTCTTATGCTGG	ACGTTGGATGTATAAACTGCCAAAGCTGGG	106
rs5030062	ACGTTGGATGCTAAATGTGCAAACCCAAGG	ACGTTGGATGAGCTAGTCTCCAAAATGACC	99
rs146922325	ACGTTGGATGGGTCTTCTGTGTCTCGTTTC	ACGTTGGATGCTCAGTGAAGTTCCCTTGTG	100
rs34290760	ACGTTGGATGCTTAAACTGAGCCTGAGCTG	ACGTTGGATGCAGGGTGGAAATTGATAGGG	120
rs139974673	ACGTTGGATGGAACCCAGTCATCTCTGAAC	ACGTTGGATGGAAGGCAATGAAACGTCCTG	103
rs10993706	ACGTTGGATGTTCATCTCTTAGCAAGGTG	ACGTTGGATGACTGTGTTTCCAGAGATGTC	120
rs8176749	ACGTTGGATGTGCTGGTCCCACAAGTACTC	ACGTTGGATGACGAGAGCCACCTGAACAA	99
rs2289252	ACGTTGGATGGATATCGCACATGTGCCAG	ACGTTGGATGGAGTTGGATGAGGAGTTAGC	97
rs2227624	ACGTTGGATGGATTCATGGGAATGTCCCG	ACGTTGGATGTTGCTGCTCATTGGCTTCTG	111
rs55823018	ACGTTGGATGTCCAGGATGGGAACTACTC	ACGTTGGATGATTGGCTGAGCTGCTGTGTG	98
rs1799963	ACGTTGGATGCCATGAATAGCACTGGGAG	ACGTTGGATGTGGAACCAATCCCGTGAAAG	122
rs2227589	ACGTTGGATGAAAGGCCTTACCCCAAGAG	ACGTTGGATGTCTCCCTGGTAGTTACAGTC	109
rs3136520	ACGTTGGATGTCCTGCACTGTGTTCTGAAG	ACGTTGGATGCAAAAAAGGGAGACCCTGTC	106
rs57615042	ACGTTGGATGCCATTGCTGAGTATACGAGG	ACGTTGGATGCACAGTCAGAATAATCTGC	117
rs169713	ACGTTGGATGCAATGCTAGCAGCTACTATC	ACGTTGGATGCTCTCAAAGCACATCATGCC	100
rs687289	ACGTTGGATGAAGTCACCTGTCACCTCTAC	ACGTTGGATGTGCCTTGGTCAGTGCAGTC	109
rs3136516	ACGTTGGATGCAAGTTCAAGGTCACATCAG	ACGTTGGATGCTGGTGAACACATCTTCTGG	110
rs6795524	ACGTTGGATGATGGGAGACCAAACACGTAG	ACGTTGGATGCTTTGGTTACGGTTTTGGC	109
rs710446	ACGTTGGATGCAGGACTCTGATCTCATTGC	ACGTTGGATGGGATATCAGGGATCCAATCG	105
rs1063856	ACGTTGGATGTGCACTCCAGGTCATAGTTC	ACGTTGGATGATGGTCAAGCTGGTGTGTC	108
rs8176719	ACGTTGGATGCATGTGCAGTAGGAAGGATG	ACGTTGGATGCGTTGAGGATGTCGATGTTG	109
rs8176743	ACGTTGGATGTAGTAGAAATCGCCCTCGTC	ACGTTGGATGAGATCCTGACTCCGCTGTTC	147
rs13146272	ACGTTGGATGGTCAGGGACTTACACTGTTG	ACGTTGGATGTGGCTTTGGCTTGATCTCTG	123
rs4524	ACGTTGGATGCTTGGGTCCCTTATGCTTAG	ACGTTGGATGTGTCACAGGGATACGTCTAC	99
rs8178847	ACGTTGGATGTGTCCCGATAGAGGGAATTG	ACGTTGGATGCCATCCATACCTACGTTTGC	93
rs1613662	ACGTTGGATGATTTCCCAGGAACCTCTGTG	ACGTTGGATGAGAATGGACCCTGCAGAAC	106
rs2036914	ACGTTGGATGGAGACAAGGAGTGCTTTGAG	ACGTTGGATGGAGATCCTAAAAAGCAGCAG	98
rs9411377	ACGTTGGATGTGACAAAGTGAGACTCCACC	ACGTTGGATGTATCCATTACTGAGAGTGGG	128
rs2227631	ACGTTGGATGACAGGAGACCAACGTGTAAG	ACGTTGGATGGGAGGAAGAGGATAAAGGAC	101
rs1801133	ACGTTGGATGCTTCACAAAGCGGAAGAATG	ACGTTGGATGCTTGAAGGAGAAGGTGTCTG	105
rs4253417	ACGTTGGATGATTTGAGACAGGGTCTTGC	ACGTTGGATGGATTGCTTGAGCCTAGGAAG	114
rs16984852	ACGTTGGATGCCAGCCCAGACACTTCTTG	ACGTTGGATGGCACTTATAAACTCGAGCCC	108
rs2066865	ACGTTGGATGGACCCCATGTTGAAAACTCC	ACGTTGGATGGTGGTGGTTTTTAATGGTC	146
rs2066864	ACGTTGGATGGCATTTTTATGACCACTTG	ACGTTGGATGACCTGGGAATTTGAAACTC	138
rs2519093	ACGTTGGATGTAACACTTCCCTTCAAAAG	ACGTTGGATGATACACCTACTATGAACCC	142
rs1799983	ACGTTGGATGACAGCTCTGCATTCAGCAC	ACGTTGGATGGGGCAGAAGGAAGAGTTC	126
GAPDH	ACGTTGGATGGAAATTAACTGGACAGGGC	ACGTTGGATGGGTTAAAATACAGCTTCCCC	121

Supplementary Table S2 Extension primer sequences for the 39 VTE-associated SNPs and GAPDH control

SNP_ID	Extension primer sequences	Extension primer Direction	UEP mass (Da)
rs78707713	GAGCAAAACAGCG	F	4002.6
rs56324901	GCATGGTGGTGCA	R	4031.6
rs60509203	CTGAATCATCTGTC	R	4214.8
rs12097293	GCTTCAAATCCAAAC	R	4506
rs5030062	AACCCAAGGCAAGAT	R	4580
rs146922325	CGCTTCTTCTCCATCC	R	4720.1
rs34290760	GCTGTCTAGAGGAAAA	F	4955.3
rs139974673	CTCTGAACCATGGCACC	F	5116.3
rs10993706	AGTATATCTACATTTAC	R	5144.4
rs8176749	GTGGGTTTGTGGCGCAG	F	5314.4
rs2289252	GGCCAGGATGAGAGGGC	R	5326.5
rs2227624	GGGCTCCCGTGACAGGTC	F	5517.6
rs55823018	CTACTCCCCCAGGTCAAGG	R	5734.7
rs1799963	AACTGGGAGCATTGAGGCT	R	5893.8
rs2227589	GGGAGAGCACTTGAAATGA	F	5926.9
rs3136520	ATCTGAAGGCACCTTTAGCA	F	6102
rs57615042	TGTTTGGTTGCTTAGACCAC	F	6115
rs169713	CTTCGCACTCTACACAGCCAG	F	6312.1
rs687289	ATGGGGCATCTCTGGACACGG	R	6488.2
rs3136516	TTCAAGGTCACATCAGTATTCC	R	6670.4
rs6795524	TGCTCTTTGACCGAGTGTTCTT	R	6699.3
rs710446	GCCTCCTATATCACCAGCTCCCA	F	6865.5
rs1063856	GTCATAGTTCTGGCACGTTTTGG	F	7077.6
rs8176719	AGTAGGAAGGATGTCCTCGTGGT	R	7160.7
rs8176743	CCTCCCGGCTGCTTCCGTAGAAGC	F	7266.7
rs13146272	GGTTGGTAAAAGTATGTAGGATCT	R	7487.9
rs4524	ATTATGCTTAGCATGTTCTTGACTT	F	7629
rs8178847	GTTTCCAGCTGATGGCTTATAAACA	F	7657
rs1613662	GCCGGTTACCAACAGAACCACCTTCC	R	7846.1
rs2036914	TTGAGGGTAATTGAATCATTTCAAGG	R	8065.3
rs9411377	CCAAAGTGAGACTCCACCTCAAAAAAA	F	8224.4
rs2227631	AGGAGACCAACGTGTAAGTTTCACTTC	R	8284.4
rs1801133	GAGAAAAGCTGCGTGATGATGAAATCG	F	8422.5
rs4253417	TGGTCTTGCTCTGTCACTCAGATTTGGT	F	8568.6
rs16984852	ACACTTCTTGCCGCTGCGCGCAGCCCCTG	F	8767.7
rs2066865	GTTTGTTTCCTAAGACTAGATACATGGTA	R	8922.8
rs2066864	TTTATGACCACTTGTCATTTATTTTGTCTT	R	9109.9
rs2519093	GGAAAAATGAATAAATAAGCCACCGACTGA	R	9275.1
rs1799983	GGCTGCCCCTGCTGCTGCAGGCCCCAGATGA	F	9475.1
GAPDH	AGTCTCTGTCCCTTTT	F	4147.7

Abbreviation: F, forward; R, reverse
